# The Emerging Role of p27 in Development of Diseases

**DOI:** 10.17140/CSMMOJ-4-e006

**Published:** 2018-05-09

**Authors:** Qiwei Yang, Ayman Al-Hendy

**Affiliations:** Department of Obstetrics and Gynecology, University of Illinois at Chicago, Chicago, IL 60612, USA

## INTRODUCTION

The cell cycle regulation and tumor suppressor p27 encoded by *CDKN1B* plays a key role in many cellular events.^1–3^ p27 is a member of the Cip/Kip family of cyclin-dependent kinase (CDK) inhibitors, which functions to negatively regulate cell cycle progression at the G1/S boundary in response to antiproliferative stimuli. In addition, numerous p27 functions, not related to CDK inhibition, have been described. For instance, cytosolic p27 plays a role in the regulation of cytoskeleton assembly/disassembly, therefore, regulates the cell morphology and movement. In addition, p27 is involved in apoptosis and autophagy modulation.^4–6^

Mutations, abnormal expression and mislocalization of p27 have been found in many diseases suggesting the important role of p27 in the pathogenesis of diseases. Human p27 gene (*CDKN1B*) was cloned in 1994^7^ and mapped to chromosome 12p13. Later on, p27 mutations were discovered in several types of human cancers including breast cancer, sporadic parathyroid adenomas, endocrine neoplasia, small intestine neuroendocrine tumors.^2,8–14^

Several types of tumors show decreased expression of p27, including breast, colon, esophageal carcinomas, head and neck cancers, hematological tumors lung, prostate, melanomas and ovarian tumors.^1,15^ The decreased expression of p27 is due to increased proteasome-mediated protein degradation, correlates with poor prognosis of patients. Several other studies demonstrate that a decrease in the expression levels of p27 protein contributes to tumor development by increasing in CDK activity and cell proliferation.^15–17^

In addition, an increased body of evidence demonstrates that mislocalization of p27 contributes to the development of aggressive phenotype and anticancer therapy resistance. p27 levels and subcellular localization are catalyzed by different kinases that modulate degradation and nuclear-cytoplasmic shuttling. In endometrial carcinoma cell lines, p27 is low and/or predominantly cytoplasmic p27 phosphorylation at T157 by AKT (protein kinase B). Treatment with an AKT inhibitor rescues the mislocalization of p27 to the cytoplasm in endometrial carcinoma cells.^18^ The mislocalization of p27 has also been identified in other types of cancers,^19–22^ suggesting that sequestration of p27 in the cytoplasm might be an alternative way to inactivate p27-associated inhibitory activity in cancers.

## p27 AND RISK OF DISEASES

Reduced expression and mislocalization of p27 have been identified as an early event in some types of diseases. A study by Mc-Campbell et al demonstrates that loss of p27 expression is an early event in the progression of endometrial carcinoma in the setting of obesity. p27 expression is severely reduced and/or mislocalized to the cytoplasm in histologically “normal” endometrial glands and endometrial complex hyperplasia with atypia from obese women (CAH) as compared to normal weight women. In luteal phase endometrium, p27 expression is high and primarily nuclear. In contrast, in the majority of endometrial CAH, p27 expression is severely reduced or absent in >70% of these early lesions, and is harshly reduced or absent in 89% of primary endometrial carcinoma. These data indicate that loss of p27 is retained as a feature of early (CAH) and neoplastic endometrial lesions arising in the setting of obesity.^18^ Similar findings are observed in other types of human cancers.^1,23^ p27 is reduced in premalignant and non-invasive cancerous lesions, including ductal carcinoma *in situ* of the breast. The reduced p27 expression is prognostic for subsequent development of oral squamous carcinoma. In addition, in benign prostatic hypertrophy and low malignant potential of ovarian tumors, the p27 expression levels are decreased compared to normal tissues.

## p27 AS A PREDICTOR OF TREATMENT RESPONSES

For animal study, Eker rats carrying a defect in the *Tsc2* tumor suppressor gene are a genetically-defined model for endometrial hyperplasia that processes to endometrial carcinoma by 16 months of age.^18^ At the early stage of this model, appearing “pre-hyperplastic” glands with activated mTORC1 signaling correlate with loss of the wild-type *Tsc2* allele. Early life exposure to xenoestrogen accelerates the development of endometrial hyperplasia in adult female rats.^24^ Similar to human disease, loss of p27 occurs early in association with the development of obesity-associated endometrial hyperplasia. The energy balance intervention study by McCampbell et al demonstrates that caloric restriction is capable of reducing weight, providing a favorable to leptin/adiponectin ratio, and decreasing the circulating insulin levels in response to early life exposure to genistein. Importantly, caloric restriction also significantly decreases hyperplasia incidence with increased p27 expression levels and relocalization of p27 to the nucleus.^18^

In human, the effect of chemotherapy can also be predicated according to the expression levels of p27 in some types of cancers. For instance, in non-small cell lung cancer^25^ and ovarian cancers,^26^ decreased expression of p27 correlates with reduced survival in response to platinum-based chemotherapy. In breast cancer,^27^ decreased expression of p27 is associated with poor outcome after adjuvant chemotherapy. In head and neck squamous cell carcinomas,^28^ p27 expression serves as a significant predictor of chemotherapy response in multivariate analysis.

## FUTURE DIRECTIONS

Although progresses have been made to understand the role of p27 in the pathogenesis of diseases, there remains a gap in our knowledge regarding the abnormal expression and subcellular localization of p27, which contribute to the pathogenies of varied diseases. How these events link to the processes of abnormal cell cycle and development of diseases related to the network of signaling pathways and epigenome? What is the role of p27 in favorable and unfavorable effects of chemotherapy? Also, more pre-clinical studies are needed to determine the effect of treatments in varied types of cancers and diseases. For instance, the energy balance intervention study shows a potent inhibitory effect on hyperplasia incidence in Eker rat model. In addition to endometrial hyperplasia, Eker rats are also a genetically-defined model for the development of uterine fibroids.^29,30^ Does this dietary intervention also work for uterine fibroids through the same mechanism? Further understanding the mechanism and role of p27 may lead to the development of novel treatment options against many challenging diseases.
